# Leveraging the lessons learned from financing HIV programs to advance the universal health coverage (UHC) agenda in the East African Community

**DOI:** 10.1186/s41256-019-0118-y

**Published:** 2019-09-13

**Authors:** Henry Zakumumpa, Sara Bennett, Freddie Ssengooba

**Affiliations:** 1 Makerere University, School of Public Health, Kampala, Uganda; 2 Johns Hopkins University, Bloomberg School of Public Health, Baltimore, USA

## Abstract

**Background:**

Although there is broad consensus around the need to accelerate progress towards universal health coverage (UHC) in Sub-Saharan Africa, the financing strategies for achieving it are still unclear. We sought to leverage the lessons learned in financing HIV programs over the past two decades to inform efforts to advance the universal health coverage agenda in the East African Community.

**Methods:**

We conducted a literature review of studies reporting financing mechanisms for HIV programs between 2004 and 2014. This review is further underpinned by evidence from a mixed-methods study entailing a survey of 195 health facilities across Uganda supplemented with 18 semi-structured interviews with HIV service managers.

**Results:**

Our data shows that there are six broad HIV financing strategies with potential for application to the universal health coverage agenda in the East African Community (EAC); *i*) *Bi-lateral and multi-lateral funding vehicles:* The establishment of HIV-specific global financing vehicles such as PEPFAR and The Global Fund heralded an era of unprecedented levels of international funding of up to $ 500 billion over the past two decades *ii) Eliciting private sector contribution to HIV funding:* The private sector’s financial contribution to HIV services was leveraged through innovative engagement and collaborative interventions *iii) Private sector-led alternative HIV financing mechanisms:* The introduction of ‘VIP’ HIV clinics, special ‘HIV insurance’ schemes and the rise of private philanthropic aid were important alternatives to the traditional sources of funding *iv) Commodity social marketing:* Commodity social marketing campaigns led to an increase in condom use among low-income earners *v) The use of vouchers:* Issuing of HIV-test vouchers to the poor was an important demand-side financing approach *vi) Earmark HIV taxes:* Several countries in Africa have introduced ‘special HIV’ taxes to boost domestic HIV funding.

**Conclusions:**

The lessons learned from financing HIV programs suggest that a hybrid of funding strategies are advisable in the quest to achieve UHC in EAC partner states. The contribution of the private sector is indispensable and can be enhanced through targeted interventions towards UHC goals.

## Background

Universal health coverage (UHC) is gaining increasing importance as a global health priority [1]. In 2015, the Sustainable Development Goals (SDGs) enshrined the attainment of UHC by 2030 in the new international development agenda [2]. According to the World Health Organization (WHO), universal health coverage is ‘the single most powerful concept public health has to offer’ [3]. Several countries in Sub-Saharan Africa which include Kenya, Rwanda and Zimbabwe have launched plans for achieving UHC [4].

Although there is broad consensus around the need for accelerating progress towards attaining UHC, the ways and means of achieving are still unclear [5, 6]. It has been estimated that low and middle-income countries face a $ 274 billion ‘financial UHC gap’ [7]. Hence, devising financing strategies for realizing the UHC agenda, especially in low-income and middle-income countries is critical [7, 8].

The emerging global trend towards regional integration and countries coalescing around larger blocks such as the European Union (EU) or South African Development Community (SADC), presents unique opportunities for collaboration and harmonization of UHC strategies across multiple countries with similar socio-economic characteristics and history [9–11].

### The East African community (EAC)

The East African Community (EAC) is a block of six countries that comprise Kenya, Uganda, Tanzania, Rwanda, Burundi and South Sudan [12].The treaty establishing the EAC was signed in November 1999 and came into force in July 2000 [13]. The EAC has a combined population of 172 million people and a Gross Domestic product (GDP) of US $ 172 billion [14]. EAC countries aspire to have a common market and a single customs union. Beyond the imperative of economic cooperation, article 108 of the treaty setting up the EAC community calls for harmonized ‘national health policies that promote quality health in the community’ [1]. EAC countries share many characteristics especially with regard to population health, weak health systems and a shared colonial legacy [15]. In the EAC Region, only about 65% of health care financing comes from domestic sources (through governments, the private sector and out-of-pocket spending) with over 35% provided through international assistance [16]. In addition, out-of-pocket spending in the region ranges between 10 and 30% compared with a WHO threshold of 20% [16]. Given these similarities across partner states, coordination and cooperation of efforts to advance the UHC agenda within the region is a shared priority echoed in the EAC motto ‘One people, one destiny’. Indeed, in 2016, a joint communique from the ministers responsible for health and finance in EAC partner countries affirmed commitment towards achieving universal health coverage (UHC) [17]. Kenya roll-outed out a universal health coverage pilot in the western city of Kisumu in December 2018 while Rwanda operates a long-standing community-based health insurance scheme. In June 2019, the Uganda cabinet approved a national health insurance scheme [18, 19].

Over the past two decades, there has been accumulating evidence documenting innovations in financing HIV programs that could be leveraged upon in efforts to advance the UHC agenda in EAC countries [20–22]. In seeking to leverage HIV lessons for promoting the universal health coverage agenda in the EAC, we reflect on the UHC goals of ensuring financial risk protection, expanding health coverage and access to quality health products and services [1–4].

### A convergence around UHC and HIV response goals

**Fig. 1:**
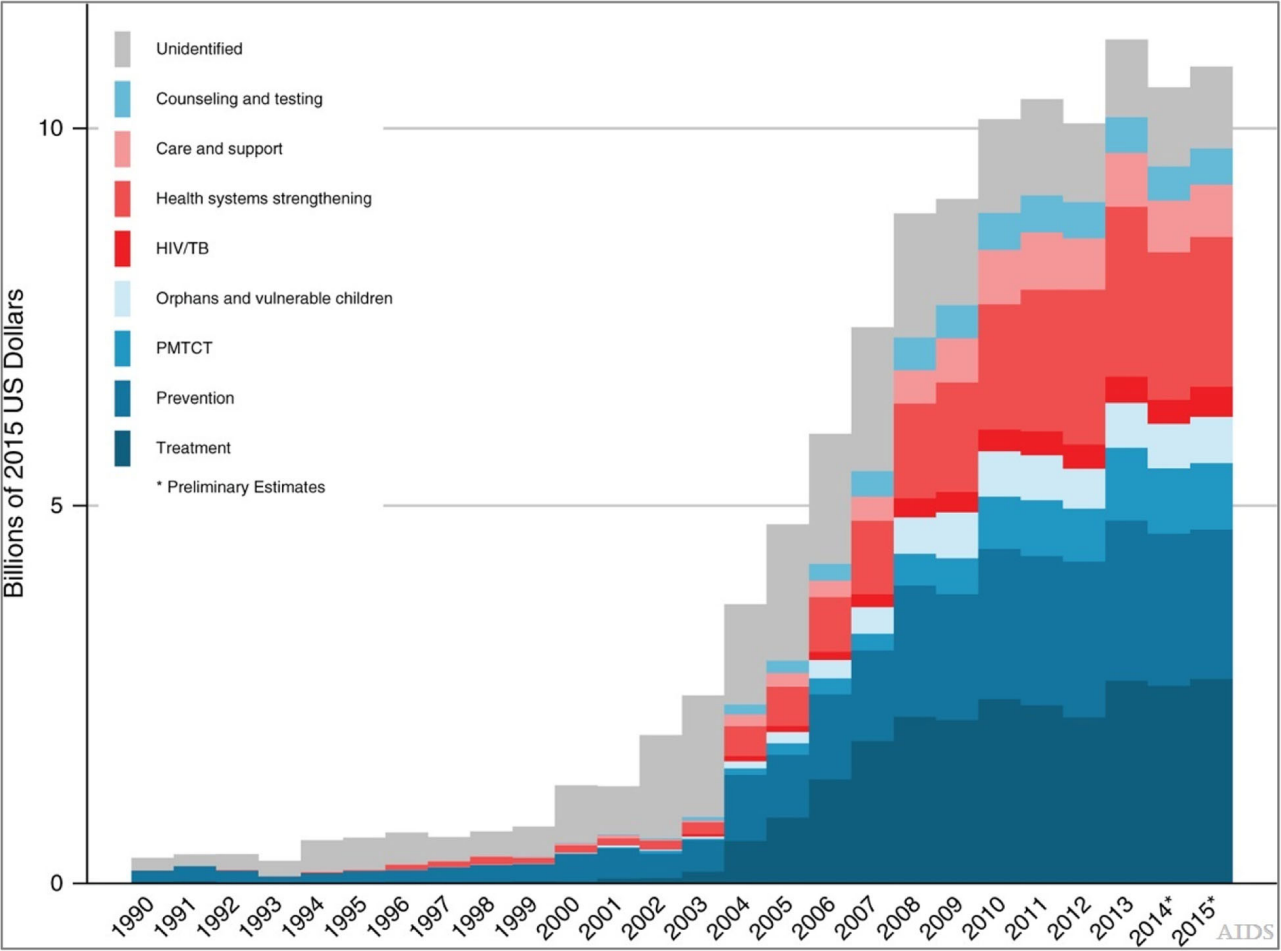
Tracking development assistance for HIV/AIDS: the international response to a global epidemic

t has been acknowledged that UHC and the global HIV response share similar goals of expanding service coverage, attention to marginalized populations and the poor [20]. Others have argued that implementing UHC can directly support HIV services scale-up as has been demonstrated in South Africa and Thailand where HIV services have been directly funded from national UHC pools [21]. Conversely, HIV-specific donor funding in low-income countries such as in Ethiopia where 35,000 community health workers were recruited to strengthen HIV services at the primary care level can synergize non-HIV services such as malaria control, maternal and child health and combating Non-Communicable Diseases (NCDs) [23]. Broadly, donor HIV funding such as PEPFAR investments in health workforce recruitments and strengthening pharmaceutical supply chains as well as infrastructure support synergizes non-HIV services such as malaria control [20]. On the other hand, it has been observed that vertical donor HIV funding could detract from broader health-systems goals such as in Ghana where expenditure on anti-retroviral therapy (ART) was funded outside the national health insurance pool [20]. Figure 1 shows the proportion of HIV funding that goes to HIV treatment. Other scholars such as McIntyre and colleagues [8] have discussed ways in which the fragmentation associated with disease-specific funding vehicles can be ameliorated by building more towards UHC goals. Proposals in this direction have included mounting calls for improving the integration of HIV services into general health systems and maximizing the synergies between HIV funding and broader health systems strengthening [24, 25].

We drew from three inspirations for this review. We drew upon the notion that the international HIV response is a *path finder* for global health, a call re-echoed at the 22nd International AIDS Conference in Amsterdam in July 2018 [25]. Our objective was to draw upon the literature relating to HIV financing innovations over the past two decades, to inform financing strategies for realizing the UHC agenda in the East African Community (EAC).

## Methods

### Literature review

This review is informed by a literature review conducted as part of a broader four-year doctoral research study examining the financing strategies for promoting the sustainability of HIV programs in Uganda [26]. This review is also underpinned by evidence from a mixed-methods study involving a survey of nearly 200 health facilities across Uganda and 18 semi-structured interviews with HIV service managers [26]. For the literature review component, we broadly followed the steps recommended for conducting a literature review proposed by McKee and colleagues [27]. Firstly, we searched PubMed, Web of Science, Science Direct, MEDLINE and Google scholar using a simple search strategy aimed at identifying studies conducted on financing OR funding mechanisms/strategies for sustaining HIV OR AIDS programs in low-income countries which were published between 2004 and 2014. The 2004 start date was selected because this marked the initial national roll-out of HIV services scale-up in many low-income countries [26]. Secondly, we searched web sites of development agencies, and international financing institutions (e.g. World Bank, Kaiser Family Foundation, Institute for Health Metrics and Evaluation (IHME) for published reports on HIV financing sources and approaches (final search conducted August 2017). Our literature search specifically focused on two core components of HIV programs; a) anti-retroviral therapy (ART) OR HIV treatment and b) HIV prevention OR testing AND funding OR financing strategies. Our exclusion criteria were the following: an opinion piece; an editorial; an abstract meeting; and the link between HIV programs and financing mechanism or strategy is not clear. The articles identified from our literature review were scrutinized for relevance as guided by Kutzin’s [6] framework(s) on health financing mechanisms (*risk pooling, revenue raising, purchasing and benefits*) which served as a thematic framework for a qualitative content analysis we conducted of our search results. We situate these health financing mechanisms within the universal health coverage goal of people receiving the health services they need without suffering financial hardship such as by reducing dependence on out-of-pocket expenditure and increasing the size of risk pools [8]. A third source of this review was our invited participation at an international conference on health financing for Universal Health Coverage in low and middle-income countries held in Kampala, Uganda in August of 2017 under the European Union-funded SPEED project (http://speed.musph.ac.ug/symposium/). We merged the findings emerging from all the three sources of our review and categorized them under the six themes presented in the Results section.

## Results

### Bi-lateral and multi-lateral HIV funding vehicles

**Fig. 2:**
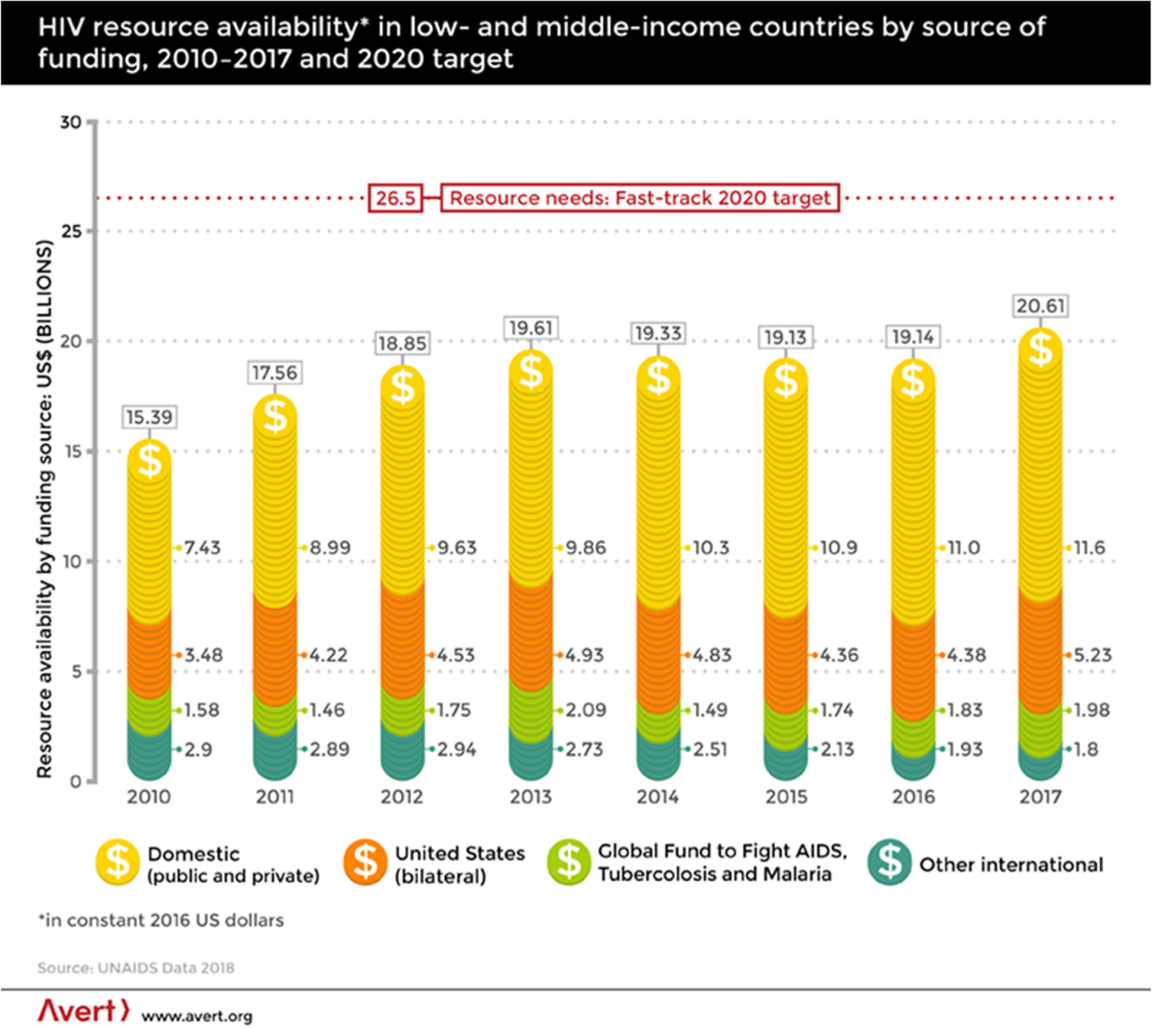
HIV resource availability in low and middle income countries

The unprecedented international mobilization of funding for HIV services scale-up in SSA since 2003 presents lessons that can be leveraged upon to advance the UHC agenda in EAC [25]. The establishment of bilateral and multilateral funding schemes for the global HIV response such as the Global Fund for AIDS, Malaria and Tuberculosis established in 2002 and that of the Presidents’ Emergency Plan for AIDS Relief (PEPFAR) commissioned in 2003 are worthy lessons to draw upon in the quest to achieve universal health coverage and highlights the potential for resource mobilization from international funding sources for global health (See Fig. 2) [26, 28]. The Global Fund for instance, is an international financial organization which is estimated to account for over 20% of all international HIV funding through periodic contributions from countries such as Norway, Germany, France and Italy [29]. The Global Fund makes grant decisions based on applications from donor countries with an increasing focus on low-income countries. This follows a recent trend of ‘graduating’ middle-income countries such as Peru from its aid programs. In all, Global Health Initiatives (GHIs) such as PEPFAR and the Global Fund are said to have mobilized over $ 500 billion for the global HIV response over the past two decades [30].

Of the six EAC countries, four (Kenya, Uganda, Tanzania, Rwanda and Burundi) are among the 15 PEPFAR focus-countries [31]. The idea of setting up a global fund for UHC implementation in SSA modeled on the Global Fund (GFAMT) has been mooted [32]. At the level of EAC, a commitment by member countries to broadly increase the budget for health was made in 2016. There is need to renew this commitment in the context of mounting calls for implementing UHC globally.

### Eliciting the private sector’s contribution to HIV funding

From the perspective of governments and donors, the private sector (especially for-profit providers) has been driven to cater to the segment of the population with the ‘ability to pay’ which leaves the majority poor unreached. As such, there have been mounting calls for devising interventions and policy responses aimed at integrating the private sector in the drive to attain the Universal Health Coverage (UHC) targets enshrined in the new sustainable development goals (SDGs) agenda [1, 33]. The private sector is characterized by heterogeneity and incorporates private for-profit, private not-for-profit and traditional medicine practitioners [34]. The range of players is diverse indeed and extends from large corporate hospitals to informal drug sellers [1, 33–35].

The calls for policy responses targeting the private sector stem from the notion of a systemic ‘market failure’ in meeting demand for health care and the need for remedial or corrective measures aimed at promoting Universal Health Coverage [35]. The private sector is said to have comparative advantages (over government) in health service delivery in some respects which ought to be leveraged upon [1, 33, 35]. For instance, select faith-based providers are often based in parts of countries where government presence is weak. Typically, these are remote or hard-to-reach areas not served by public infrastructure such as tarmac roads and the national electricity grid [34]. Additionally, faith-based providers and non-state actors such as MSF (medicines san frontiers) often have more experience (and are often better resourced) in mounting emergency epidemic and disaster responses. Non-profits such as Marie Stopes have accumulated experience and expertise in family planning services in EAC partner states which governments in this region can support and build upon [35]. With regard to the for-profit sector, large corporate hospitals often are able to mobilize substantial private financing for expensive medical equipment and technology such as those used in advanced treatments of cancers and heart diseases [35]. Hence, public-private partnerships are critical in leveraging additional financing for achieving UHC and the health sector in general [1, 33, 35].

In Uganda, USAID sought to secure the private sector’s contribution towards the costs of HIV service delivery through a counter-part funding scheme that enabled for-profit clinics to commence provision of HIV services starting in 2009 [36]. To this end, a memorandum of understanding was signed with selected for-profit clinics in Uganda coupled with multiple follow-up stakeholder planning meetings. USAID then provided on-site support to these for-profit clinics to enable them qualify for ART site-accreditation by the Ministry of Health in Uganda by providing them with medical equipment and health workforce trainings there by expanding the national network of HIV treatment sites across Uganda including in parts where state presence was particularly weak [36–38]. In 2017, the World Bank published a report that assessed the potential of engaging the private sector in Uganda towards attainment of UHC goals in Uganda [38]. In the majority of countries in Africa, the private sector constitutes more than half of all health service providers [33–35]. Due to this dominant role in service provision, there is increasing consensus that the private sector is too important a sector to be ignored in the efforts to realize universal health coverage [38, 39]. There is immense potential of building upon these private sector engagements in Uganda by USAID and the World Bank through diffusing them across the broader East African Community region in efforts to achieve UHC goals of expanding access to health services.

### Commodity social marketing

Commodity social marketing involves adopting commercial marketing techniques that create demand for quality products such as condoms or family planning commodities whose quality has been assured through sustained advertising and other demand-creation campaigns [40]. Commodity social marketing is one of the commonest interventions for reaching the poor (and those with limited information) through subsidized pricing of quality health products [33, 41, 42]. According to Montagu and colleagues [33], social commodity marketing offers lessons in successful condom distribution through the private sector both as a health financing vehicle but also for improving health product quality. A study which reviewed Demographic Health Survey data across Sub-Saharan Africa between 1998 and 2007 concluded that subsidized pricing of condoms in networks of for-profit clinics resulted in increased condom use including among low-income earners [33, 43]. More importantly for UHC aspirations, with regard to condom use, commodity social marketing has been shown to improve equity in access, across gender, in a multi-country study [43].

### The use of vouchers in HIV prevention

Voucher schemes have been defined as ‘a type of demand-side consumer-led near-cash social transfer that can be redeemed for goods and services’ [44]. Vouchers have been found to be an efficient subsidy for vulnerable sub-populations especially the poor [33]. A study conducted in the United States between July and September 2013, reported that the provision of vouchers for an oral HIV test kit redeemable at a network of pharmacies in Los Angeles improved HIV testing among socially-disadvantaged groups and their subsequent linkage to HIV care [45]. In Bangladesh, a voucher scheme for HIV testing targeting men who have sex with men (MSM) implemented between 2014 and 2015 revealed a high rate of utilization of vouchers at 89% [46]. This study reported a median turn around voucher redemption rate of 7 days suggesting that vouchers promise high utilization rates in low and middle-income countries and have promising application relevance in the East African Community. Additionally, this study showed that the voucher scheme improved HIV testing (by 76%), as well as linkage to care [46, 47].

Elsewhere, voucher schemes or programs have been used to support poor expectant mothers to deliver in a private health facilities in Rwanda, Uganda and Zambia [33]. Leveraging the East African Community framework especially through the committee of Ministers of Health, vouchers could be applied to a range of issues including maternal and child health (MNCH) and are an area worthy of consideration as we seek ways of operationalizing UHC in the East African Community partner states.

### Private sector-led innovations in financing HIV services

A study in Uganda, documents private sector-led innovations in devising alternative financing mechanisms for anti-retroviral therapy (ART) programs between 2004 and 2014 [26]. These innovations have been key in diversifying funding away from a heavy dependence on traditional partners such as PEPFAR and The Global Fund [26]. These innovations include the introduction of ‘VIP’ or ‘Executive’ HIV clinics which cater to ‘higher-tier’ clients and accordingly charge higher service fees for upper middle class Ugandans in an after-hours clinic model implemented at the Infectious Diseases Institute (IDI) clinic in Kampala in November 2013 [48, 49]. The higher service charges for these ‘higher-tier’ recipients of care are then ploughed back to support poorer HIV patients [49]. Furthermore, a section of for-profit clinics in Uganda introduced a private ‘special HIV insurance’ scheme for patients that is based on a paid annual premium that offers ‘umbrella’ coverage for all HIV-associated costs for insured clients during the year [26]. The expanding coverage of employer-provided private medical insurance schemes in Uganda and the broader East African Community presents opportunities of raising new financing for health services beyond the traditional sources [26] and is a welcome trend that supplements public finance sources for achieving UHC. A 2017 study in Uganda documents the increasing reliance by private not-for-profits (PNFPs) on funding from private foundations and individuals as supplemental financing for HIV programs [26]. Private philanthropic donations emerged as an important source of supplemental funding for ART programs which remains a largely untold story [26]. The majority of philanthropic organizations, faith-based organizations and private individuals were from North America and Western Europe which suggests the existence of good will for increased investments in health in the East African Community from non-bilateral sources from the west that could be leveraged upon to expand access to health services especially those targeting the poor and vulnerable. This paper [26] reveals important non-GHI funders such as Africa Health Care Foundation (AHF) based in California, USA which was reported as the most important funder of health facilities providing ART a large part of South-Western Uganda with a relatively HIV burden [26].

### The rise of earmark taxes for HIV

**Fig. 3:**
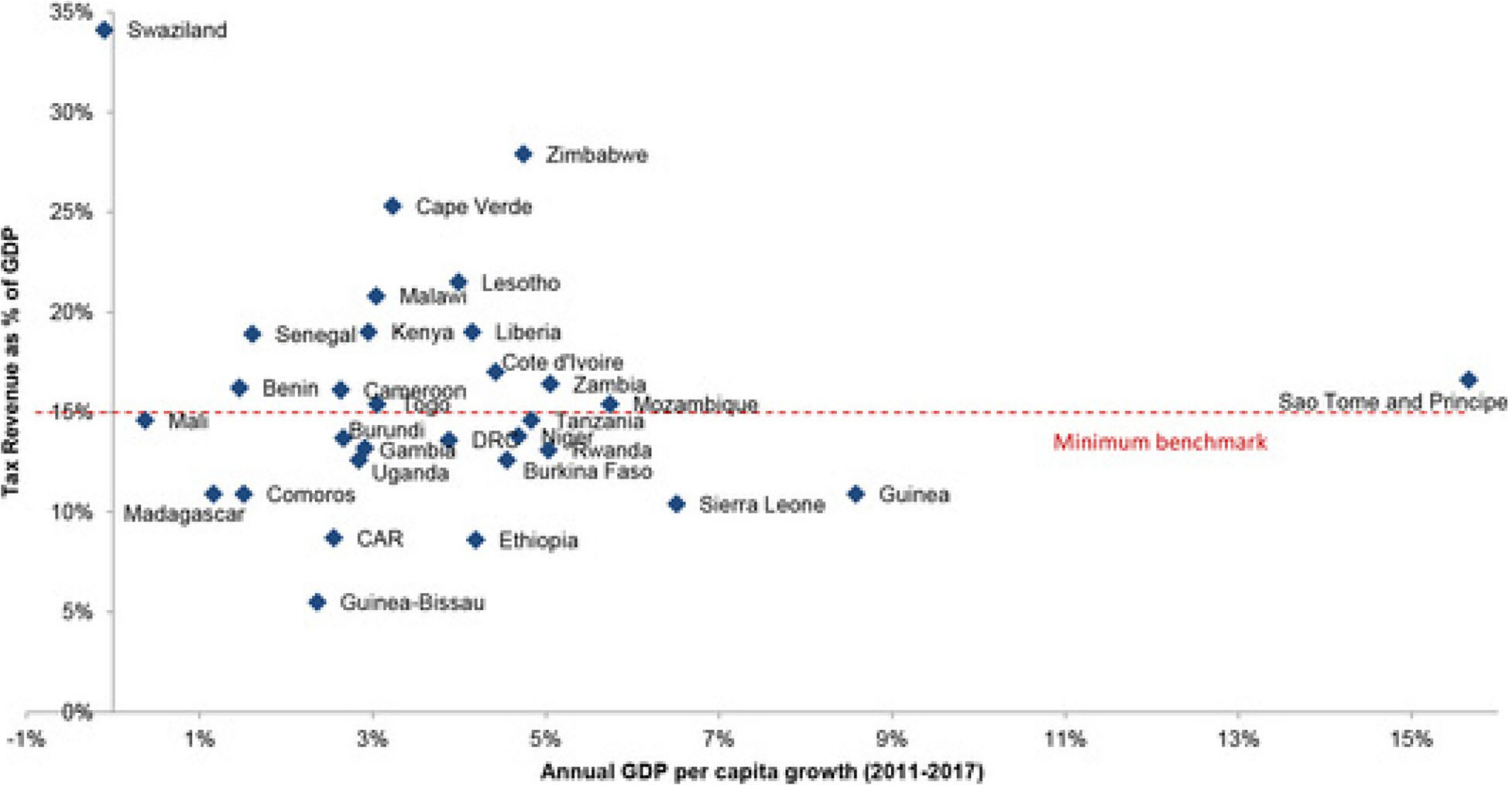
Growth and taxation rates in low- and lower middle-income countries

**Fig. 4:**
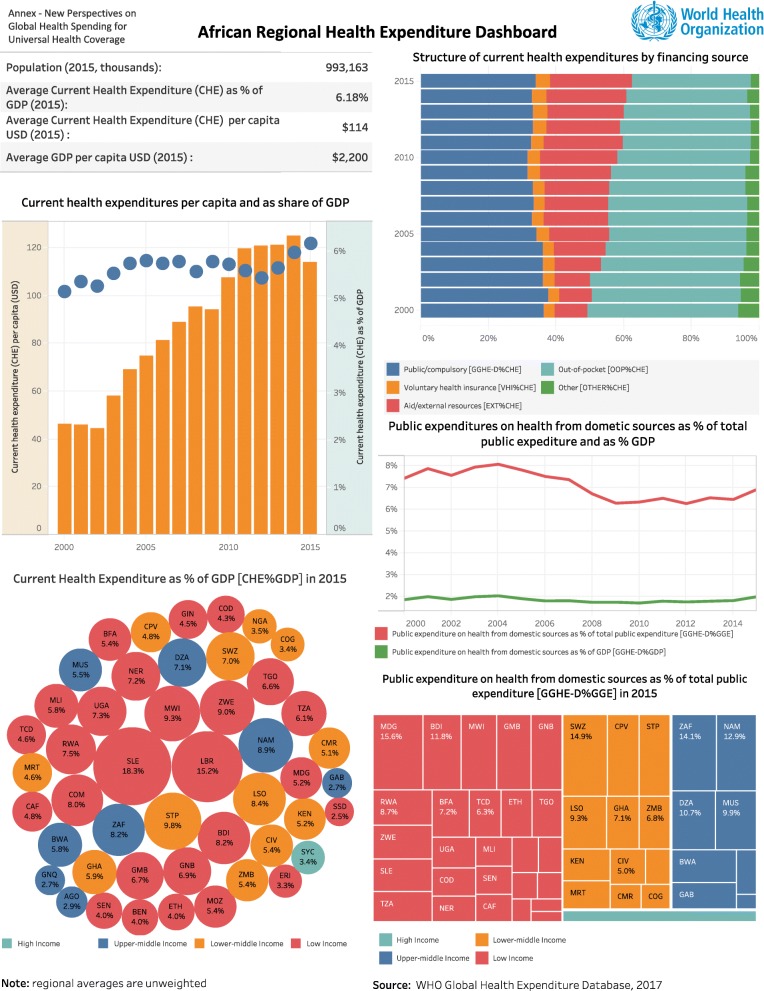
African Regional Health Expenditure Dashboard

In response to calls for increased national ownership of HIV programs, several governments in Africa have introduced earmark taxes for HIV causes [50–52]. In Zimbabwe, an ‘AIDS levy’ was introduced in 2000 in form of a 3% tax on businesses and the formal sector workforce to support the national HIV response [53, 54]. On 18th December 2008, Ivory Coast introduced a ‘solidarity’ tax on tobacco products which goes into the National AIDS Fund (FNLS) for funding that country’s national HIV response. Uganda enacted a law establishing the national AIDS Trust Fund (ATF) in July 2014 [26]. The AIDS Trust Fund will be supported by a tax on soft drinks in Uganda and presents the promise of increasing country ownership of HIV programs which are currently highly dependent on international assistance by as much as 85% [26]. As Fig. 3 shows, broadly there is immense potential for increasing tax revenue for achieving UHC and health causes in general within EAC partner states and there exist opportunities for realizing this under a common EAC framework [55]. Figure 4 is a dashboard that shows WHO statistics on public expenditure on health from domestic sources as a percentage of total public expenditure in the WHO-AFRO region in 2015. Of the EAC partner countries represented in this dashboard on African Regional Health Expenditure’, Rwanda (RWA) is shown to have the highest expenditure at 7.8% revealing ample room for expanding the fiscal space for public spending on health within the EAC region based on the Abuja declaration threshold of 15%. Although we highlight the role of special HIV levies, they are not restricted to national governments in Africa. On the global stage, over the past five years, UNITAID which was established in 2006 has been funded by up to 50% through an ‘air ticket’ levy in 10 countries which set this levy according to contributor country specifications [54, 56].

## Conclusions

Lessons learned from HIV financing suggest that a hybrid of funding schemes are advisable in the quest to achieve universal health coverage in the East African community. From a public finance perspective, there are calls for increasing the fiscal space for implementing UHC from national budgets. The private sector is indispensable in efforts to realize UHC and should be co-opted through targeted interventions but also with regard to private sector-led innovations in health financing health services. Leveraging the lessons of HIV financing also suggests that tapping into the growing middle class in East Africa to help contribute to financing of health services in the context of UHC.

## Data Availability

Not applicable.

## Abbreviations

AIDS: Acquired Immune Deficiency Syndrome
ART: Antiretroviral therapy
ARV: Antiretroviral
ARVs: Anti-retrovirals
GFATM: Global Fund to Fight HIV/AIDS, Tuberculosis and Malaria
HIV: Human Immunodeficiency Virus
MOH: Ministry of Health
NMS: National Medical Stores
OIs: Opportunistic infections
PEPFAR: United States President’s Emergency Plan for AIDS Relief
SSA: Sub-Saharan Africa
UNAIDS: The Joint United Nations Program on HIV/AIDS

## References

[1] McPake B, Hanson K (2016). Managing the public–private mix to achieve universal health coverage. Lancet.

[2] Mukherjee JS, Mugunga JC, Shah A, Leta A, Birru E, Oswald C, Jerome G, Almazor CP, Satti H, Yates R, Atun R (2019). A practical approach to universal health coverage. Lancet Glob Health.

[3] WHO. Promoting health through the life course. Retrieved 3 Mar 2019 from https://www.who.int/life-course/news/events/uhc-day/en/

[4] Sambo LG, Kirigia JM (2014). Investing in health systems for universal health coverage in Africa. BMC Int Health Hum Rights.

[5] Mills A, Ataguba JE, Akazili J, Borghi J, Garshong B, Makawia S, Mtei G, Harris B, Macha J, Meheus F, McIntyre D (2012). Equity in financing and use of health care in Ghana, South Africa, and Tanzania: implications for paths to universal coverage. Lancet.

[6] Kutzin J (2012). Anything goes on the path to universal health coverage? No. Bull World Health Organ.

[7] Stenberg K, Hanssen O, Edejer TT, Bertram M, Brindley C, Meshreky A, Rosen JE, Stover J, Verboom P, Sanders R, Soucat A (2017). Financing transformative health systems towards achievement of the health sustainable development goals: a model for projected resource needs in 67 low-income and middle-income countries. Lancet Glob Health.

[8] McIntyre D, Garshong B, Mtei G, Meheus F, Thiede M, Akazili J, Ally M, Aikins M, Mulligan JA, Goudge J (2008). Beyond fragmentation and towards universal coverage: insights from Ghana, South Africa and the United Republic of Tanzania. Bull World Health Organ.

[9] Marten R, McIntyre D, Travassos C, Shishkin S, Longde W, Reddy S, Vega J (2014). An assessment of progress towards universal health coverage in Brazil, Russia, India, China, and South Africa (BRICS). Lancet.

[10] UNDP. Regional Integration and Human Development: A Pathway for Africa. 2011. Retrieved 4 Mar 2019 from: https://www.undp.org/content/dam/undp/library/Poverty%20Reduction/Trade,%20Intellectual%20Property%20and%20Migration/RIR%20English-web.pdf

[11] Te V, Griffiths R, Law K, Hill PS, Annear PL (2018). The impact of ASEAN economic integration on health worker mobility: a scoping review of the literature. Health Policy Plan.

[12] Bachmann V, Sidaway JD (2010). African regional integration and European involvement: external agents in the East African community. S Afr Geogr J.

[13] Rugera SP, McNerney R, Poon AK, Akimana G, Mariki RF, Kajumbula H, Kamau E, Mpawenimana S, Said SY, Toroitich A, Ronoh W (2014). Regulation of medical diagnostics and medical devices in the East African community partner states. BMC Health Serv Res.

[14] EAC. East African Community. One people, One Destiny. An overview of EAC. Retrieved 13 Apr 2019 from: https://www.eac.int/overview-of-eac

[15] Katembo B. Pan Africanism and Development: the East African community model. Journal of Pan African Studies. 2008;2(4).

[16] World Bank WB. Building integrated markets within the East African community: EAC opportunities in public-private partnership approaches to the region's infrastructure needs: The World Bank; 2014.

[17] EAC: Joint communique of the Minister of Health and Minister of Finance on universal health and HIV& AIDS in the EAC. Retrieved 6 Feb 2019 from: https://www.eac.int/communique/487-759-23-joint-communique-of-the-ministers-of-health-and-ministers-of-finance-from-the-east-african-community-partner-states-high-level-ministerial-dialogue-meeting-on-sustainable-financing-for-universal-health-and-hiv-aids-coverage-for-the-eac-region

[18] WHO. Kenya rolls out Universal Health Coverage. Retrieved 8 Mar 2019 from: https://www.afro.who.int/news/kenya-rolls-out-universal-health-coverage.

[19] The Independent. Retrieved 8 Jul 2019 from: https://www.independent.co.ug/national-health-insurance-scheme-bill-approved-by-cabinet/

[20] Jay J, Buse K, Hart M, Wilson D, Marten R, Kellerman S, Odetoyinbo M, Quick JD, Evans T, Piot P, Dybul M (2016). Building from the HIV response toward universal health coverage. PLoS Med.

[21] Buse Kent, Jay Jonathan, Odetoyinbo Morolake (2016). AIDS and universal health coverage—stronger together. The Lancet Global Health.

[22] Ministry of Health. Kenya. Leveraging the HIV response to drive Universal Health Coverage in Kenya.Retrieved 8 Jan 2019 from: https://nacc.or.ke/wp-content/uploads/2018/09/LEVERAGING-THE-HIV-RESPONSE-TO-DRIVE-UNIVERSAL-HEALTH-CARE-IN-KENYA-2ND.pdf

[23] Rabkin M, El-Sadr WM (2011). Why reinvent the wheel? Leveraging the lessons of HIV scale-up to confront non-communicable diseases. Global public health.

[24] Yu D, Souteyrand Y, Banda MA, Kaufman J, Perriëns JH (2008). Investment in HIV/AIDS programs: does it help strengthen health systems in developing countries?. Glob Health.

[25] Bekker LG, Alleyne G, Baral S, Cepeda J, Daskalakis D, Dowdy D, Dybul M, Eholie S, Esom K, Garnett G, Grimsrud A (2018). Advancing global health and strengthening the HIV response in the era of the sustainable development goals: the international AIDS society—lancet commission. Lancet.

[26] Zakumumpa H, Bennett S, Ssengooba F (2017). Alternative financing mechanisms for ART programs in health facilities in Uganda: a mixed-methods approach. BMC Health Serv Res.

[27] McKee M, Britton A (1997). Conducting a literature review on the effectiveness of health care interventions. Health Policy Plan.

[28] Soni A, Gupta R (2009). Bridging the resource gap: improving value for money in HIV/AIDS treatment. Health Aff.

[29] Warren AE, Wyss K, Shakarishvili G, Atun R, de Savigny D (2013). Global health initiative investments and health systems strengthening: a content analysis of global fund investments. Glob Health.

[30] Hecht R, Flanagan K, Huffstetler H, Yamey G (2018). Donor transitions from HIV programs: what is the impact on vulnerable populations?.

[31] El-Sadr WM, Holmes CB, Mugyenyi P, Thirumurthy H, Ellerbrock T, Ferris R, Sanne I, Asiimwe A, Hirnschall G, Nkambule RN, Stabinski L (2012). Scale-up of HIV treatment through PEPFAR: a historic public health achievement. Journal of acquired immune deficiency syndromes (1999).

[32] Reeves A, Gourtsoyannis Y, Basu S, McCoy D, McKee M, Stuckler D (2015). Financing universal health coverage—effects of alternative tax structures on public health systems: cross-national modelling in 89 low-income and middle-income countries. Lancet.

[33] Montagu D, Goodman C, Berman P, Penn A, Visconti A (2016). Recent trends in working with the private sector to improve basic healthcare: a review of evidence and interventions. Health Policy Plan.

[34] Ssennyonjo A, Namakula J, Kasyaba R, Orach S, Bennett S, Ssengooba F (2018). Government resource contributions to the private-not-for-profit sector in Uganda: evolution, adaptations and implications for universal health coverage. Int J Equity Health.

[35] Montagu D, Goodman C (2016). Prohibit, constrain, encourage, or purchase: how should we engage with the private health-care sector?. Lancet.

[36] Zakumumpa H, Bennett S, Ssengooba F (2016). Accounting for variations in ART program sustainability outcomes in health facilities in Uganda: a comparative case study analysis. BMC Health Serv Res.

[37] USAID*.* The health initiatives for the private sector (HIPS) project*.* Final Evaluation Report*,* January 2013*.* http://pdf.usaid.gov/pdf_docs/Pdacu928.pdf

[38] World Bank. Uganda Private Sector Assessment in Health: Exploring Partnership Opportunities to Achieve Universal Health Access. Retrieved on 4 Feb 2019 from https://www.globalfinancingfacility.org/uganda-private-sector-assessment-health-exploring-partnership-opportunities-achieve-universal-health

[39] Feeley F, Connelly P, Rosen S (2007). Private sector provision and financing of AIDS treatment in Africa: current developments. Current HIV/AIDS Reports.

[40] Sweat MD, Denison J, Kennedy C, Tedrow V, O'Reilly K (2012). Effects of condom social marketing on condom use in developing countries: a systematic review and meta-analysis, 1990-2010. Bull World Health Organ.

[41] Price N (2001). The performance of social marketing in reaching the poor and vulnerable in AIDS control programmes. Health Policy Plan.

[42] Rimal RN, Creel AH (2008). Applying social marketing principles to understand the effects of the radio diaries program in reducing HIV/AIDS stigma in Malawi. Health Mark Q.

[43] Chapman S, Jafa K, Longfield K, Vielot N, Buszin J, Ngamkitpaiboon L, Kays M (2012). Condom social marketing in sub-Saharan Africa and the Total market approach. Sex Health.

[44] Yam CH, Liu S, Huang OH, Yeoh EK, Griffiths SM (2011). Can vouchers make a difference to the use of private primary care services by older people? Experience from the healthcare reform programme in Hong Kong. BMC Health Serv Res.

[45] Marlin RW, Young SD, Bristow CC, Wilson G, Rodriguez J, Ortiz J, Mathew R, Klausner JD (2014). Piloting an HIV self-test kit voucher program to raise serostatus awareness of high-risk African Americans. Los Angeles BMC Public Health.

[46] Ahmed S, Ahmed F, Tazreen M, Uddin Z, Rahman A, Oyediran KA, Oyewale TO (2016). The use of vouchers in HIV prevention, referral treatment, and care for young MSM and young transgender people in Dhaka, Bangladesh: experience from ‘HIM’initiative. Curr Opin HIV AIDS.

[47] Bassett IV, Wilson D, Taaffe J, Freedberg KA (2015). Financial incentives to improve progression through the HIV treatment cascade. Curr Opin HIV AIDS.

[48] Kakaire T, Schlech W, Coutinho A, Brough R, Parkes-Ratanshi R (2016). The future of financing for HIV services in Uganda and the wider sub-Saharan Africa region: should we ask patients to contribute to the cost of their care?. BMC Public Health.

[49] Twimukye A, King R, Schlech W, Zawedde FM, Kakaire T, Parkes-Ratanshi R (2017). Exploring attitudes and perceptions of patients and staff towards an after-hours co-pay clinic supplementing free HIV services in Kampala. Uganda BMC health services research.

[50] UNAIDS. Zimbabwe AIDS levy generates new resources for treatment. 2012. Retrieved 8 Mar 2019 from: http://www.unaids.org/en/resources/presscentre/featurestories/2012/february/20120221zimbabwe

[51] Oberth G, Whiteside A (2016). What does sustainability mean in the HIV and AIDS response?. Afr J AIDS Res.

[52] Anyanwu JC, Siliadin YG, Okonkwo E (2013). Role of fiscal policy in tackling the HIV/AIDS epidemic in southern Africa. Afr Dev Rev.

[53] Bhat N, Kilmarx PH, Dube F, Manenji A, Dube M, Magure T (2016). Zimbabwe's national AIDS levy: a case study. SAHARA-J: Journal of Social Aspects of HIV/AIDS.

[54] Vassall A, Remme M, Watts C, Hallett T, Siapka M, Vickerman P, Terris-Prestholt F, Haacker M, Heise L, Haines A, Atun R (2013). Financing essential HIV services: a new economic agenda. PLoS Med.

[55] Yates R (2015). Universal health coverage: progressive taxes are key. Lancet.

[56] Hecht R, Bollinger L, Stover J, McGreevey W, Muhib F, Madavo CE, de Ferranti D (2009). Critical choices in financing the response to the global HIV/AIDS pandemic. Health Aff.

